# Maximizing treatment response in patients with neuropathic-subtype postural orthostatic tachycardia syndrome

**DOI:** 10.3389/fneur.2026.1796563

**Published:** 2026-05-26

**Authors:** Ryan G. Rilinger, Adeline Y. Chin, Christopher Cantrell, Mackaleigh Levine, Samantha J. Stallkamp Tidd, Robert Wilson

**Affiliations:** 1 Cleveland Clinic Lerner College of Medicine, Cleveland, OH, United States; 2 Department of Neurology, Cleveland Clinic Foundation, Cleveland, OH, United States; 3 Department of Neuromuscular Medicine, Cleveland Clinic Foundation, Cleveland, OH, United States; 4 Department of Neurology, University of Rochester Medical Center, Rochester, NY, United States

**Keywords:** ivabradine, neuropathic subtype, postural orthostatic tachycardia syndrome, POTS, treatment

## Abstract

**Background:**

Postural Orthostatic Tachycardia Syndrome (POTS) is notoriously difficult to diagnose and successfully treat. POTS encompasses several different subtypes, but little research attention has focused on optimizing the diagnosis and treatment of specific subtypes. This study aims to identify variables potentially predictive of neuropathic POTS and determine which current medical therapies for POTS are most successful in the neuropathic population.

**Methods:**

Single-center retrospective review identified 382 patients diagnosed with POTS between 2018 and 2020. Diagnosis was established via the head-up tilt table test; neuropathic subtype was established via skin biopsy or quantitative sudomotor axon reflex test. Logistic regression identified variables predictive of the neuropathic subtype; Cox proportional hazard and Kaplan-Meier estimator modeling were employed to compare treatment response to medications between neuropathic and non-neuropathic patients.

**Results:**

Patients with neuropathic POTS treated with ivabradine exhibited a median treatment response duration of 26.7 months in the neuropathic group vs. 10.7 months in the non-neuropathic group, reflecting a significantly reduced risk of treatment failure (hazard ratio = 0.273, *p* = 0.028). No other studied treatments demonstrated different response. Of the clinical and demographic variables studied, only elevated baseline heart rate was significantly associated with the neuropathic POTS subtype, though with questionable clinical significance.

**Discussion:**

Patients with neuropathic POTS may exhibit different medication treatment response compared to patients with non-neuropathic POTS, though prospective studies are needed for confirmation. Further study to identify optimal treatment regimens for different subtypes of POTS is a promising direction to improve the quality of care for patients with POTS.

## Introduction

Postural Orthostatic Tachycardia Syndrome (POTS) is an understudied and undertreated autonomic nervous system disorder with patients often experiencing multi-year delay to diagnosis and repeat treatment failures (1, 2). Patients with POTS may be classified into neuropathic, hyperadrenergic, and hypovolemic subtypes based on various clinical parameters, such as elevated plasma norepinephrine in the hyperadrenergic subtype or the low renin and aldosterone in the hypovolemic subtype (3). These subtypes, however, do not differ in their clinical presentation, meaning additional testing is required to characterize each patient's subtype (4). A handful of prior studies have suggested POTS subtypes might respond differently to available medical therapies, but as a result of limited quantity of research on POTS, consensus guidelines for treatment selection by subtype do not yet exist (5, 6).

Testing necessary to characterize neuropathic POTS commonly includes methods to identify small-fiber neuropathy, such as nerve fiber density skin biopsy or quantitative sudomotor axon reflex testing (QSART) (7–9). However, these tests can be expensive, time-consuming, and invasive; additionally, there is a similar lack of consensus on guidelines for diagnostic test selection. Consequently, many patients with POTS receive substantial delay to diagnosis (or no diagnosis at all) of a specific POTS subtype, and additionally struggle to receive therapy tailored to their unique disease presentation (10). Frustration with delays in symptom relief can alienate patients with POTS from the healthcare system, further elevating the risk of poor outcomes. Frequent medication changes can elevate patient stress through shifting side effects and fluctuating medication cost, as well as increase provider strain if insurance paperwork is required for medication coverage.

Little is known about other clinical factors that may predict the neuropathic subtype, muddying the waters for clinicians who must decide whether or not to recommend the burden of additional subtype-specific testing for their patients with POTS (10).

For patients with symptoms uncontrolled by conservative measures such as exercise or compression garments, clinicians must select from a wide variety of medications when treating POTS—therapies commonly prescribed for the treatment of POTS vary widely in mechanism of action and target of effects. Common pharmacologic mechanisms, with examples provided in parentheses, include mineralocorticoid agonists (fludrocortisone), medications to decrease heart rate (beta-blockers, ivabradine), vasoconstrictors (midodrine), and prescribed salt intake (11). Literature is sparse regarding the efficacy of available medical therapies between subtypes of POTS, but the data available suggests different POTS subtypes may very well respond differently to certain medications. In one example, patients diagnosed with neuropathic POTS demonstrated greater control of symptoms with midodrine than patients with hyperadrenergic POTS (12). The clinical picture for clinicians managing POTS is therefore complicated two-fold: consensus guidance is lacking regarding the identification of POTS subtypes and the subsequent application of tailored therapy.

In this retrospective review, we aim to explore clinical factors predictive of the neuropathic POTS subtype, as well as probe medication response between patients with neuropathic and non-neuropathic POTS. Through this analysis, we hope to identify potential preferred medications for physicians to select based on patient subtype.

## Materials and methods

### Ethical standards

This study has been approved by the appropriate ethics committee, The Cleveland Clinic Institutional Review Board (IRB), and has therefore been performed in accordance with the ethical standards laid down in the 1964 Declaration of Helsinki and its later amendments. The IRB granted a waiver of informed consent for this retrospective chart review study protocol.

### Participants

We retrospectively identified 382 patients diagnosed with POTS via the head-up tilt table test (HUTT), a diagnostic tool with demonstrated higher sensitivity for POTS diagnosis than basic orthostatic vital signs at the Cleveland Clinic between 2018 and 2020 (13). A diagnosis of POTS was confirmed by HUTT when the following criteria, identified by Stallkamp-Tidd et al. (2), were met:

1. Sustained elevation in heart rate of at least 30 beats per minute above the patient's baseline within the first 10 min of upright tilt.
2. Absence of sustained orthostatic hypotension (a decrease in systolic blood pressure of at least 20 mmHg or a decrease in diastolic blood pressure of at least ten mmHg from the patient's baseline) between 3 and 10 min of upright tilt.
3. Symptoms of orthostatic intolerance present for at least 3 months preceding the initial visit.
4. Diagnosis of POTS confirmed by a physician.
5. All other medical causes for tachycardia or orthostatic intolerance ruled out.

Of the initial pool of 382,256 (67%) underwent additional testing with either intraepidermal nerve fiber density skin biopsy (IENFD; through which reduced nerve fiber density evidence via punch biopsy is suggestive of small fiber neuropathy) or the quantitative sudomotor axon reflex test (QSART; low scores indicate abnormally low sweat response to electric stimulation of the skin and are considered suggestive of small autonomic nerve fiber damage) to identify presence or absence of the neuropathic subtype of POTS. Given the lack of consensus guidelines regarding the application of small-fiber neuropathy testing in POTS, IENFD, and/or QSART testing were performed at provider discretion, typically in response to patient-reported symptoms suggestive of small-fiber neuropathy (e.g., numbness, paresthesias) (14, 15). Patients were classified as having the neuropathic subtype of disease if they scored at or below the fifth percentile for either IENFD or QSART (16–18). Cutoff thresholds for these two tests are provided in Supplementary Table 1.

### Data collection

Electronic medical records were queried for relevant demographic factors, including age, sex, and race, and clinical factors including number of lines of therapy, time to POTS diagnosis, duration of response to therapy (defined as the time from initial medication prescription until decision to discontinue therapy for uncontrolled symptoms and/or intolerable adverse effects), presence of neurological findings on physical examination, response to patient questionnaires, cardiovascular parameters, and presence of additional comorbidities. The standardized neurological exam applied to patients with autonomic dysfunction in our center includes an assessment of cranial nerve function, a detailed sensory exam (with light touch, pinprick, vibration, and proprioceptive modalities), a detailed motor exam, assessment of reflexes, assessment of coordination, and assessment of gait. To confirm duration of response to therapy, follow-up visit notes and prescription records were reviewed to confirm active receipt of medication. As this is a retrospective analysis, medications were selected at physician discretion in accordance with standard-of-care; treatments were not assigned to participants, and participants were permitted to receive more than one therapy at a time. This analysis did not include medication doses or order of therapies received (i.e., this analysis does not distinguish whether treatments were first-line vs. subsequent lines of therapy). Laboratory tests and clinical biomarkers were not included.

Patients who had ongoing response to their current therapy at the time of data collection were censored; patients who were lost to follow-up were censored at date of last contact. As a retrospective review, follow-up cadence was determined by physician discretion and standard-of-care; the typical cadence at our institution is follow-up visits at 6-month intervals. Cardiovascular parameters included baseline heartrate as well as change in heartrate during the HUTT. Included comorbidities were Ehlers-Danlos Syndrome, gut dysmotility, and autoimmune disease; each patient evaluated at our institution for workup of POTS receives a standard review-of-systems and past medical history to assess for these specific conditions. For this study, a provider-entered diagnosis in the medical record was required to determine positivity. Patient self-assessment of subjective disease experience was collected using the National Institute of Health's Patient Reported Outcome Measurement Information System (PROMIS) physical and mental health T-scores; assessment of mental health was collected via the Generalized Anxiety Disorder 7-item scale (GAD-7) and the Patient Health Questionnaire 9-item scale (PHQ-9) (19–21).

### Statistical analysis

Analysis was performed only on the 256 patients who underwent testing for presence or absence of neuropathic disease. All numerical variables were assessed for normality with the Shapiro-Wilk test and for homogeneity of variances with the Levene test. Mean with standard deviation, or median with interquartile range, were reported for normally and non-normally distributed variables, respectively. Categorical variables were characterized as proportions. Descriptive statistics were applied to the neuropathic and non-neuropathic subtypes separately. Comparisons between these two groups were conducted via Welch's *t*-test for normally distributed continuous variables or the Mann-Whitney U-test for non-normally distributed continuous variables; categorical variables were compared via the chi-square test unless expected variable frequencies were less than five, in which case Fisher's exact test was applied. Logistic regression was employed to evaluate associations between clinical variables (treatment course, HUTT parameters, prior diagnoses, neurologic examination findings, and patient questionnaires) and subsequent diagnosis of neuropathic POTS. Responses to nine POTS-directed treatments were compared between patients with and without the neuropathic subtype via Cox proportional hazard and Kaplan-Meier estimator modeling; the latter was also used to produce median treatment response duration with interquartile range to account for subject censoring in these parameters. The primary outcome was time to treatment failure, defined as the number of months between beginning a medication and discontinuing the therapy due to either uncontrolled symptoms or intolerable side effects. Response to desmopressin and droxidopa were not analyzed given very low sample size: two recipients of droxidopa, one recipient of desmopressin.

For Table 1, the current formatting makes it appear as if the rows “Time to POTS diagnosis” and “Treatment courses (per person-year)” are sub-categories within “Race”, which is not accurate. We would suggest moving these two rows to the top of the table, below “Age” and above “Sex”, so that they do not appear to be part of another category.

## Results

The patient sample was characterized with descriptive statistics (Table 1). No significant differences were observed between patients with the neuropathic and non-neuropathic subtypes of POTS for demographic characteristics of age, sex, and race. Similarly, the groups did not differ in their time to POTS diagnosis, number of treatment lines received (adjusted per person-year), or the frequency with which they received different lines of therapy.

**Table 1:** Patient and clinical characteristics.

Characteristic	Neuropathic POTS (*n* = 145)	Non-neuropathic POTS (*n* = 111)	*p-value*
	Statistic	Statistic	
**Age, median (IQR)**	30.0 (24.0–40.0)	29.9 (22.5–36.0)	0.091^*^
**Time to POTS diagnosis**	32.23 (12.3, 79.2)	32.35 (13.9, 71.8)	0.781^*^
**Treatment courses (per person-year)**	2.27 (1.13, 3.03)	1.95 (0.69, 2.88)	0.122^*^
Sex			
Female	132 (91.0)	102 (91.9)	0.808^†^
Male	13 (9.0)	9 (8.1)	
Race			
Black	5 (3.45)	3 (2.70)	0.649^‡^
White	134 (92.4)	100 (90.1)	
Other	6 (4.14)	8 (7.21)	
Treatments received			
Beta-blockers	54 (37.2)	56 (50.5)	0.132^†^
Desmopressin	1 (0.07)	3 (2.70)	0.335^‡^
Droxidopa	0 (0.00)	2 (1.80)	0.209^‡^
Fludrocortisone	33 (22.7)	27 (24.3)	0.874^†^
Ivabradine	13 (8.97)	12 (10.8)	0.820^†^
Midodrine	36 (24.8)	31 (27.9)	0.937^†^
Pyridostigmine	32 (22.1)	18 (16.2)	0.110^†^
Salt tablets	17 (11.7)	13 (11.7)	0.764^†^
Neuropathic test modality			
IENFD only	8 (5.5)	22 (19.8)	< 0.001^‡^
QSART only	94 (64.8)	79 (71.2)	
Both IENFD and QSART	43 (29.7)	10 (9.0)	

“Statistic” is “No. (%)” unless noted otherwise. All statistics are representative of the full patient sample with neuropathic testing available (n = 256). IEFND, intraepidermal nerve fiber density, QSART, quantitative sudomotor axon reflex test.

^*^Mann-Whitney U-test, ^†^chi-square test, ^‡^Fisher's exact test.

Logistic regression modeling of demographic and clinical patient variables identified only median baseline heart rate associated significantly with neuropathic POTS (75 beats per minute in the neuropathic group vs. 72 beats per minute in the non-neuropathic group, *p* = 0.021) (Table 2). Maximum change in heart rate during the HUTT did not differ between the subtype groups. When comparing patients with and without abnormal findings on their neurological examination, patients with abnormal findings in their neurological examination were less likely (21.4% of patients with neuropathic subtype compared to 32.4% of patients with non-neuropathic subtype, *p* = 0.049) to have neuropathic POTS. No other comorbidities studied (EDS, gut dysmotility, autoimmune disease) differed in prevalence between the subtype groups. Patient self-assessment of subjective disease experience (via PROMIS physical and mental health T-scores) and mental health (via GAD-7 and PHQ-9) did not differ between the subtype groups either.

**Table 2:** Regression comparison of clinical variables with POTS subtype.

Numerical parameter	Neuropathic POTS (*n* = 145)	Non-neuropathic POTS (*n* = 111)	Coefficient	*p-value*
Cardiovascular				
Baseline HR	**75 (68, 82)**	**72 (61.5, 81)**	**4.01**	**0.021**
HUTT change in HR	43 (37, 49)	41 (36, 49)	0.39	0.788
Treatment course				
Time-to-diagnosis (months)	32.2 (12.3, 79.2)	32.3 (13.9, 71.8)	4.15	0.648
Lines of therapy (PPY)	2.27 (1.13, 3.02)	1.95 (0.69, 2.88)	0.24	0.310
Patient experience				
PROMIS physical health	34.9 (29.6, 42.3)	37.4 (29.6, 39.8)	−0.08	0.954
PROMIS mental health	39.9 (31.3, 45.8)	41.1 (35.0, 45.8)	−0.98	0.482
GAD-7	4 (1, 8)	6.5 (2.5, 9.75)	−1.14	0.467
PHQ-9	10 (5, 13)	9 (6, 14)	0.01	0.991
**Co-morbidities**	**Neuropathic POTS (*****n*** = **145)**	**Non-neuropathic POTS (*****n*** = **111)**	**Odds ratio**	***p-value***
Ehlers-Danlos syndrome	25 (17.2)	20 (18.0)	−0.01	0.872
Gut dysmotility	76 (52.4)	63 (56.7)	−0.04	0.491
Autoimmune disease	46 (31.7)	39 (35.1)	−0.03	0.568
Abnormal neurological exam	**31 (21.4)**	**36 (32.4)**	**-0.17**	**0.049**

Clinical variable data stratified by patients with and without neuropathic POTS. For numerical parameters, median (IQR) and linear regression with coefficient is provided; for co-morbidities, count (percent) and logistic regression with odds ratio is provided.

HR, heart rate; HUTT, head-up tilt table testing; PPY, per person-year.

Bolded entries are statistically significant, p < 0.05.

Patients with neuropathic POTS had significantly reduced risk of treatment failure when treated with ivabradine (HR = 0.273, *p* = 0.028) vs. the non-neuropathic patients (Table 3).

**Table 3:** Cox proportional hazard models for duration of treatment response.

Medication	Number of treatment failures (% of recipients)	Hazard ratio (Neuropathic vs. non-neuropathic)	*p-value*
Beta-blockers	116 (59.8)	1.01 (0.646, 1.59)	0.948
Fludrocortisone	65 (67.0)	0.983 (0.475, 2.04)	0.964
Ivabradine	26 (55.3)	**0.273 (0.086, 0.867)**	**0.028**
Midodrine	62 (55.9)	0.948 (0.537, 1.67)	0.855
Pyridostigmine	48 (64.0)	1.35 (0.593, 3.08)	0.473
Salt tablets	27 (57.4)	0.843 (0.345, 2.06)	0.707

Each hazard ratio reflects the risk of experiencing treatment failure among neuropathic POTS patients compared to non-neuropathic POTS patients. 95% confidence interval is provided for each hazard ratio.

Bolded entries are statistically significant, p > 0.05.

**Figure 1:**
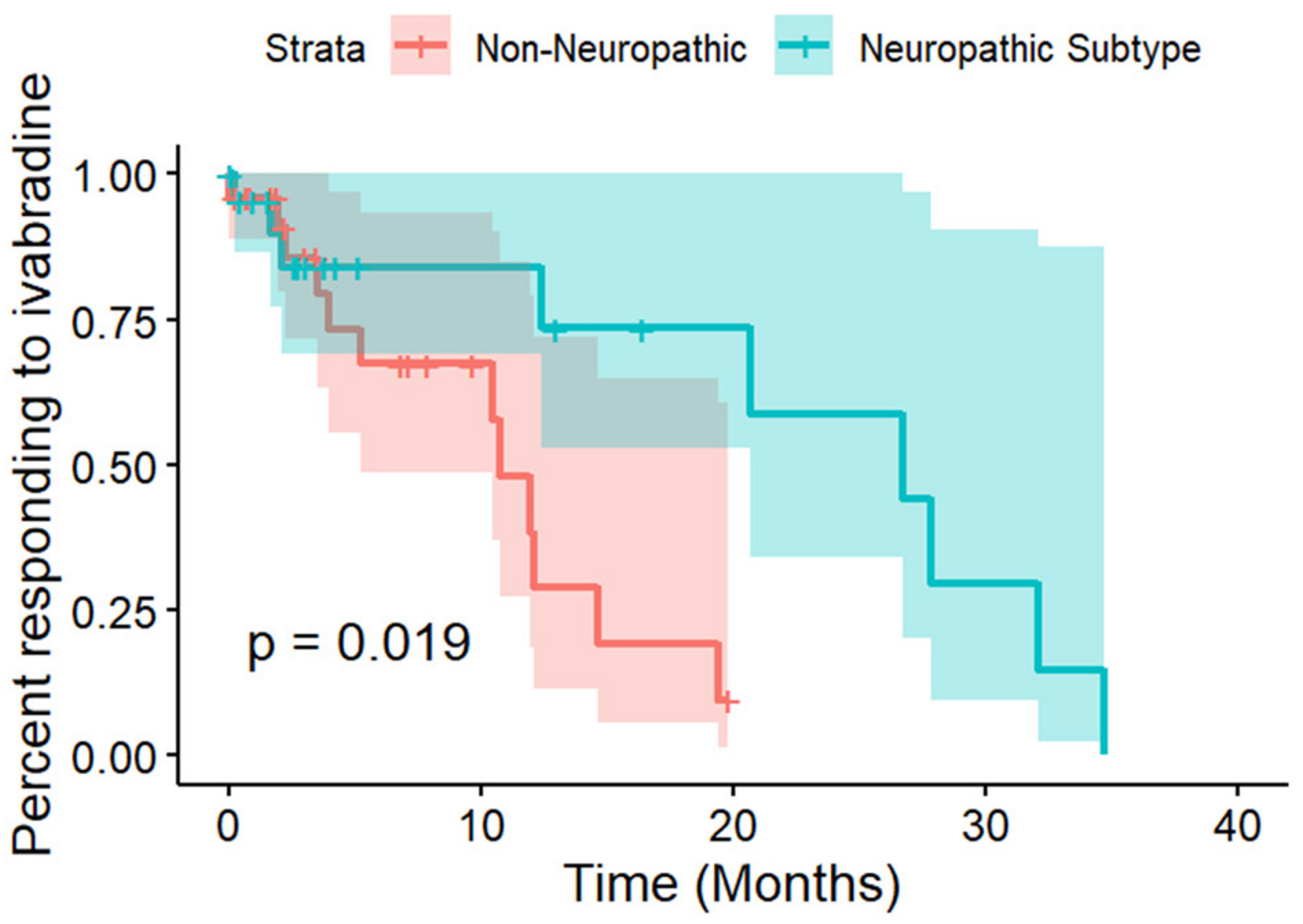
Duration of response to ivabradine therapy. Kaplan-Meier curve demonstrating the duration in months of treatment response to ivabradine, stratified by patients with and without the neuropathic subtype of POTS. Statistical significance threshold *p* < 0.05 between the subtypes. Figure created with R.

**Figure 2:**
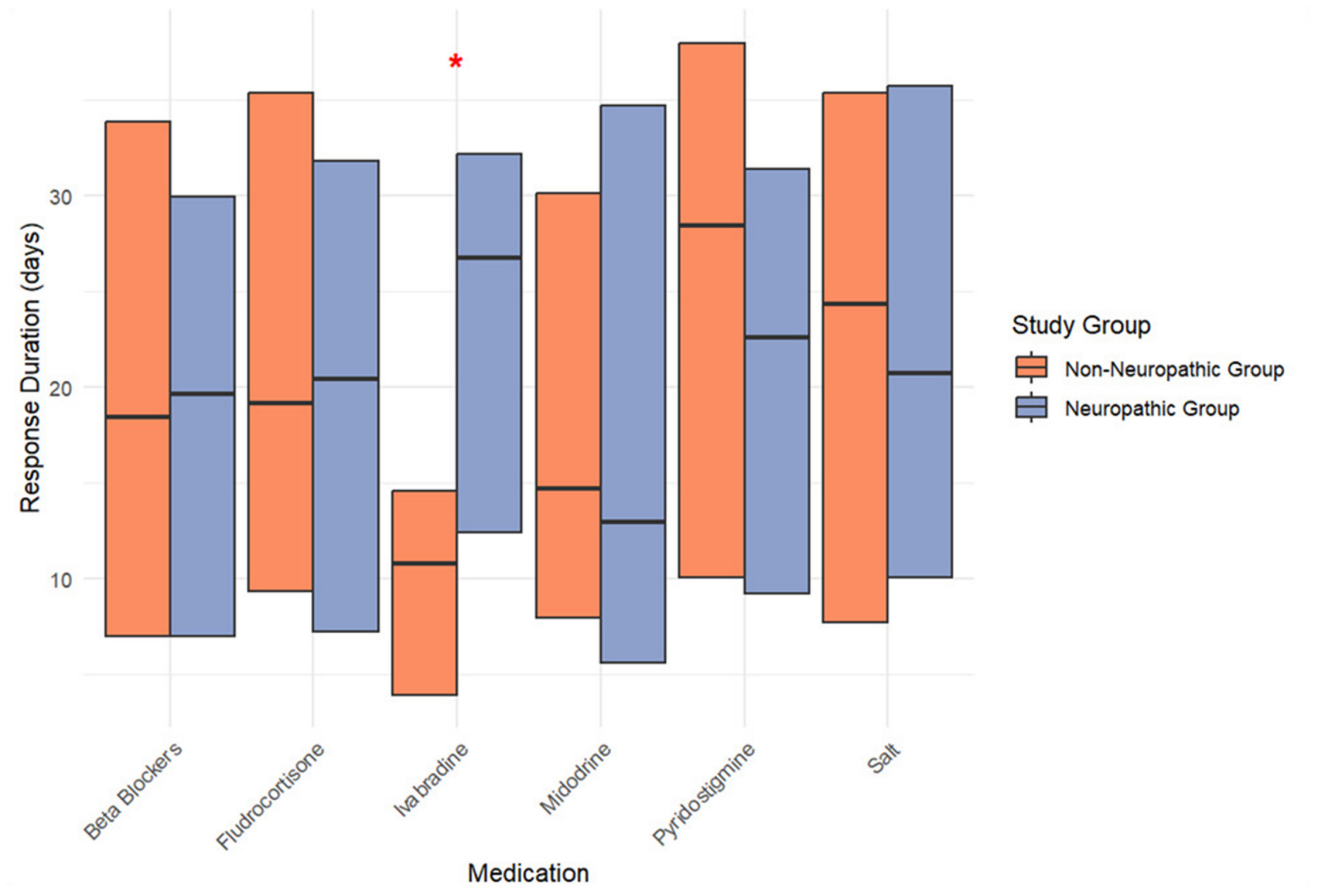
Median treatment response by therapy. Median treatment response duration and interquartile range extracted from Kaplan-Meier survival curve fit. Patients stratified by neuropathic and non-neuropathic POTS subtype. Asterisk indicates statistical significance, *p* < 0.05 between the subtypes. Figure created with R.

Median treatment duration in neuropathic patients was 26.7 months compared to 10.7 months in non-neuropathic patients (Figure 1). No significant difference in duration of treatment success between neuropathic and non-neuropathic patients was observed for any other treatment (Figure 2).

## Discussion

We present an exploratory retrospective analysis of 382 patients diagnosed with POTS, demonstrating a significantly improved response to the *I*_*F*_-channel blocking medication ivabradine among patients with neuropathic POTS compared to patients with other subtypes. Additionally, we demonstrate minimal association between demographic characteristics and clinical presentation parameters with subsequent diagnosis of neuropathic POTS, suggesting that routine history and physical exam data is likely insufficient to predict which patients with POTS carry the neuropathic subtype.

Among all medications included in this analysis, only ivabradine demonstrated improved response duration in neuropathic POTS compared to non-neuropathic disease; additionally, within the neuropathic POTS cohort, patients receiving ivabradine demonstrated longer treatment response duration than recipients of any other therapy. Ivabradine targets the sinoatrial node, selectively blocking the “funny channel” *I*_*F*_ and prolonging diastolic depolarization, slowing heart rate in dose-dependent fashion (22). Notably, in contrast to other medications affecting heart rate such as beta-blockers, ivabradine does not alter myocardial contractility and therefore has minimal impact on blood pressure. In prior studies, patients with neuropathic POTS as evidenced by abnormal QSART testing demonstrated abnormally attenuation of the expected rise in blood pressure during phase II of the Valsalva maneuver (16). If patients with the neuropathic subtype are more prone to experiencing lower blood pressures secondary to sympathetic tone impairment, they may stand to benefit more from therapy without significant blood pressure effects.

Our analysis demonstrated only higher baseline resting heart rate as a predictive factor for subsequent diagnosis of neuropathic POTS—no additional cardiovascular parameters associated with neuropathic subtype diagnosis. However, resting heart rate is unlikely to be a useful tool when selecting patients for neuropathic testing (such as skin nerve fiber biopsy or QSART) given the innumerable additional health and lifestyle factors that may affect a person's resting heart rate. Further, although statistically significant, this finding is unlikely to be clinically relevant, as the difference in median baseline heart rate differed by only three beats per minute between groups—a very thin margin that would not provide diagnostic value. Interestingly, patients with detectable neurological abnormalities on physical exam were less likely to subsequently show abnormal findings on neuropathic testing, suggesting that the small fiber neuropathy associated with neuropathic POTS does not necessarily correlate with the manifestation of testable neurological findings. Neuropathic damage may be insufficient to appear on physical examination; alternatively, patients with alternative subtypes may have been more likely to have other unrecorded comorbidities that included neurological manifestations.

Importantly, our findings center on a retrospective observation of duration of treatment response rather than objective pre- and post-treatment assessment of orthostatic vital signs or repeat tilt-table testing. In order to form definitive conclusions about medication response, prospective controlled trials in neuropathic POTS cohorts are needed; our study findings are intended to by hypothesis-generating for such future trials. Such prospective trials can also require neuropathic testing with both IENFD and QSART as eligibility criteria, or with additional modalities such as quantitative sensory testing, laser-evoked potentials, and electrochemical skin conductance, given evidence for better accuracy in the identification of small-fiber neuropathy when testing with multiple methods (7). Reported sensitivity and specificity for any specific small-fiber neuropathy test varies in the literature; combinatorial approaches in prospective studies can help combat this limitation and provide more supported diagnoses of neuropathic disease (23, 24).

As a retrospective analysis, another key limitation of this study is heterogeneity in patient treatment histories. We used IENFD and QSART to characterize neuropathic POTS, but we did not have access to needed data to identify other POTS subtypes, such as hyperadrenergic or hypovolemic disease, which may obscure interpretation of our “non-neuropathic” patient subgroup. We also relied on chart documentation, rather than prospective standardized testing, for identifying comorbidities. The majority of patients with POTS try a large number of medical therapies throughout their disease course, and as a result we cannot rule out the impact of prior therapies on the response rate of any given therapy. We also are unable to control for non-pharmacologic treatment options patients may seek in addition to their medical treatment, such as the extent of their exercise, use of compression garments, dietary salt intake, or other factors which may affect the severity of POTS symptoms. Future prospective trials could be designed to control for the use of these non-pharmacologic treatment options to better isolate medication effects. Prospective study eligibility criteria could additionally help control for patient comorbidities, disease severity, or other clinical confounders of medication response. The percent of our study sample receiving ivabradine was lower than some other included medications but reflected standard-of-care prescribing practices at our institution given the relatively higher cost of ivabradine under many patient insurance plans. Lastly, our sample size of patients receiving ivabradine was 25; this small subgroup size limits the generalizability of our findings to the broader POTS patient community, and thus our results should be considered a hypothesis-generating finding that requires confirmation in a larger, prospective, controlled study.

Knowing the long history of delay to diagnosis and frequent treatment failures suffered by this patient population, it is crucial to expand the medical community's knowledge of POTS presentation, subtypes, and available treatment options. Additional prospective controlled clinical trials taking into consideration the different subtypes of POTS are needed to build consensus regarding the optimal treatment for each subtype to minimize the patient burden incurred by repeated therapy failures. While our analysis was unable to identify patient qualities associated with the neuropathic subtype—qualities that, if known, could help clinicians more quickly recommend neuropathic testing to patients with higher pre-test probability—it is necessary to continue developing our understanding of the pathophysiology of POTS and its subtypes to inform diagnostic guidelines. Streamlining the process from initial diagnosis to selection of an effective therapy brings the medical community closer to overcoming the healthcare challenges encountered by patients with POTS.

## Data Availability

The raw data supporting the conclusions of this article will be made available by the authors, without undue reservation.
