# Supplementary material for: Maximizing treatment response in patients with neuropathic-subtype postural orthostatic tachycardia syndrome
Source: Front Neurol. 2026 May 26;17:1796563. doi: 10.3389/fneur.2026.1796563 (PMC13246704; doi:10.3389/fneur.2026.1796563)
Supplement: Supplementary file 1 [file Table_1.docx]

**Supplementary Table 1.** Positive test threshold values for intraepidermal nerve fiber test (measured in nerve fibers per millimeter) and quantitative sudomotor axon reflex test (measured in µl/cm^2^). Threshold values represent the fifth percentile for each test; patients were required to score below these threshold values on one or both testing formats to be considered positive for neuropathic subtype POTS. IENFD = intraepidermal nerve fiber test; QSART = quantitative sudomotor axon reflex test; M = value for male patients; F = value for female patients.

| **Neuropathic test modality** | **Threshold value** |
| --- | --- |
| IENFD |  |
| Proximal thigh | 7 |
| Distal thigh | 7 |
| Distal leg | 5 |
| QSART |  |
| Forearm | M: 0.76  F: 0.20 |
| Proximal leg | M: 1.27 (age 10-29), 1.10 (age 30-39), 0.93 (age 40-49), 0.75 (age 50-59), 0.58 (age 60-69) F: 0.36 (all ages) |
| Distal leg | M: 1.37 (age 10-29), 1.18 (age 30-39), 0.98 (age 40-49), 0.79 (age 50-59), 0.59 (age 60-69) F: 0.61 (age 10-29), 0.50 (age 30-39), 0.39 (age 40-49), 0.29 (age 50-59), 0.18 (age 60-69) |
| Proximal foot | M: 0.87 (age 10-29), 0.83 (age 30-39), 0.78 (age 40-49), 0.73 (age 50-59), 0.68 (age 60-69) F: 0.23 (age 10-29), 0.20 (age 30-39), 0.18 (age 40-49), 0.15 (age 50-59), 0.12 (age 60-69) |
